# Reference gene selection for quantitative gene expression analysis in black soldier fly (*Hermetia illucens*)

**DOI:** 10.1371/journal.pone.0221420

**Published:** 2019-08-16

**Authors:** Zhenghui Gao, Wenhui Deng, Fen Zhu

**Affiliations:** 1 Hubei Insect Resources Utilization and Sustainable Pest Management Key Laboratory, Huazhong Agricultural University, Wuhan, China; 2 Hubei International Scientific and Technological Cooperation Base of Waste Conversion by Insects, Huazhong Agricultural University, Wuhan, China; Gifu University, JAPAN

## Abstract

*Hermetia illucens* is an important resource insect for the conversion of organic waste. Quantitative PCR (qPCR) is the primary tool of gene expression analysis and a core technology of molecular biology research. Reference genes are essential for qPCR analysis; however, a stability analysis of *H*. *illucens* reference genes has not yet been carried out. To find suitable reference genes for normalizing gene expression data, the stability of eight housekeeping genes (including *ATP6V1A*, *RPL8*, *EF1*, *Tubulin*, *TBP*, *GAPDH*, *Actin* and *RP49*) was investigated under both biotic (developmental stages, tissues and sex) and abiotic (heavy metals, food, antibiotics) conditions. Gene expression data were analysed by geNorm, NormFinder, BestKeeper, and ΔCt programs. A set of specific reference genes was recommended for each experimental condition using the results of RefFinder synthesis analysis. This study offers a solid foundation for further studies of the molecular biology of *H*. *illucens*.

## Introduction

The black soldier fly (BSF) *Hermetia illucens* is distributed in tropical, subtropical and temperate regions of the world. BSF adults do not have functional mouthparts and therefore do not bite and transmit insect-borne diseases [1, 2]. On the contrary, BSF larvae can be used in forensic entomology to estimate the post-mortem interval [3, 4]. BSF larvae are voracious consumers and generalist saprophagous insects [5]; therefore, the larvae have been used to bio-convert a wide range of organic waste, including food processing residue, food waste, crop straw and animal manure [6–9]. The harvested larvae are exploitable for the isolation of bioactive substances (e.g., hydrolase [10, 11], antimicrobial peptides [12] and chitin [13]). Moreover, the larvae of BSF are rich in lipids and proteins, which can be used as feed or as ingredients for poultry, livestock and aquaculture [5, 14, 15]. Due to the vast economic potential of BSF, the breeding industry has developed rapidly, and research related to BSF is rapidly increasing [16, 17]. Most studies have focused on the optimization of rearing conditions and substrates for improving the nutrition and biomass of BSF larvae [18]. The molecular biology of BSF, however, is poorly researched.

Generally, real-time quantitative PCR (qPCR) is a popular and accurate technique for measuring target gene expression levels [19]. qPCR analysis is highly sensitive, and various factors, including the intrinsic variability of RNA, reverse transcription efficiency, amplification efficiency and diversity in RNA extraction methods, can affect its accuracy. Therefore, normalization is the key to achieve accurate target gene results [20, 21]. Traditionally, housekeeping genes such as *beta-actin* (*Actin*), *translation elongation factor 1-alpha* (*EF1*), *glyceraldehyde-3-phosphate dehydrogenase* (*GAPDH*), *alpha-tubulin* (*Tubulin*) and *TATA*-*binding box* (*TBP*) have been used as reference genes for the normalization of qPCR data [22, 23]. However, the expression of these reference genes fluctuates substantially under different experimental conditions [24]. A systematic study is needed to determine the appropriate reference genes for each experimental species [25].

In this study, the goal was to identify candidate reference genes in *H*. *illucens*. The stability of eight housekeeping genes under selected experimental conditions was evaluated. The candidate genes include *V-ATPase subunit A* (*ATP6V1A*), *ribosomal protein L8* (*RPL8*), *EF1*, *Tubulin*, *TBP*, *GAPDH*, *Actin*, and *ribosomal protein 49* (*RP49*). The selected environmental conditions ranged from biotic to abiotic treatments. Biotic conditions included developmental stage, tissue and sex, which were commonly used in the study of reference genes stability [23, 26, 27]. Abiotic conditions included food, heavy metals and antibiotics. These conditions will affect the conversion of BSF on waste, so many studies have focused on these conditions [8, 9, 28, 29]. As a result, a series of reference genes are recommended for gene expression studies in *H*. *illucens*.

## Materials and methods

### Insects

*H*. *illucens* samples were obtained from a colony maintained year-round in the laboratory of the Hubei International Scientific and Technological Cooperation Base for Waste Conversion by Insects, Huazhong Agricultural University (30°29′N, 114°22′E). The *H*. *illucens* colony was maintained at 27 ± 2°C, 13 L: 11 D photoperiod and 60 ± 5% relative humidity. The adults were reared in mesh cages and fed on water. Three to five blocks of three-layer corrugated cardboard were tied together with rubber bands and placed in cages to collect eggs. Eggs were held in cylindrical plastic containers (12 cm diameter, 6 cm high) with ambient humidity until eclosion. The neonatal larvae were given approximately 100 g of food (every 10 g of wheat bran was mixed with 20 ml of water), and every 48 h, (or as needed) the food was replaced. Six-day-old larvae were used in different experimental conditions. In developmental stages and during sex and tissue bioassays, the larvae continued to feed on wheat bran. In the food bioassay, 6-day-old larvae were fed wheat bran, food waste and faeces. In heavy metal and antibiotic bioassays, the 6-day-old larvae were fed wheat bran containing different concentrations of sulphonamides and cadmium.

### Experimental conditions

#### Developmental stages

The developmental stages included 0.2 g eggs, thirty 3-day-old larvae, five 8-day-old larvae, three 13-day-old larvae, three prepupae, three pupae and four adults (half male and half female).

#### Sexes

Three 1-day-old virgin male and female adults were separated and sampled.

#### Tissues

13-day-old larvae were dissected (epidermis, fat, gut, malpighian tube, haemolymph) by sterile anatomical forceps in liquid nitrogen. Tissues from ten larvae were collected and pooled as one sample.

#### Food

Thirty 6-day-old larvae were pooled in cylindrical plastic containers (12 cm diameter, 6 cm high) with 100 g of wheat bran, food waste and faeces. The diet was changed every 2 days. When 60% of the larvae became prepupae, three prepupae were taken from each sample. Death was not detected during the different food treatments.

#### Heavy metal

The diet for the larvae was wheat bran mixed with deionized water or CdCl_2_·2.5H_2_O solution. Every 10 g of wheat bran was mixed with 20 ml of solution. Finally, the concentrations of Cd were 0, 50 and 100 mg/kg dry wheat bran. Thirty 6-day-old larvae were pooled in cylindrical plastic containers (12 cm diameter, 6 cm high) with 100 g of prepared diet. The food was replaced every 2 days. When 60% of the larvae became prepupae, three prepupae were taken from each sample. Death was not detected during the heavy metal treatment.

#### Antibiotic

The concentrations of sulphonamides were 0, 50 and 500 mg/kg dry wheat bran. Thirty 6-day-old larvae were pooled in cylindrical plastic containers (12 cm diameter, 6 cm high) with a 100 g food supply. The food was replaced every 2 days. When 60% of the larvae became prepupae, three prepupae were taken from each sample. Death was not detected during the antibiotic treatment.

Sampling amount in each experimental conditions was for one biological replicate, and three biological replicates of all treatments were prepared. The experimental conditions are briefly summarized in Table 1.

**Table 1:** Experimental conditions and sampling method.

Name	Experimental conditions	Sampling period	Sampling location	Number of insects in each treatment
Developmental stages	Feeding in wheat bran without heavy metals and antibiotics	Eggs	Whole body	0.2 g
		3-day-old larvae	Whole body	30
		8-day-old larvae	Whole body	5
		13-day-old larvae	Whole body	3
		Prepupae	Whole body	3
		Pupae	Whole body	3
		Adults	Whole body	4 (2+2)
Sexes		virgin female adults	Whole body	3
		virgin male adults	Whole body	3
Tissues		13-day-old larvae	Epidermis	10
		13-day-old larvae	Fat	10
		13-day-old larvae	Gut	10
		13-day-old larvae	Malpighian tube	10
		13-day-old larvae	Haemolymph	10
Food	Wheat bran (CK)	Prepupae	Whole body	3
	Waste food	Prepupae	Whole body	3
	Faeces	Prepupae	Whole body	3
Heavy metal	0 mg/kg (CK)	Prepupae	Whole body	3
	50 mg/kg	Prepupae	Whole body	3
	100 mg/kg	Prepupae	Whole body	3
Antibiotic	0 mg/kg (CK)	Prepupae	Whole body	3
	50 mg/kg	Prepupae	Whole body	3
	500 mg/kg	Prepupae	Whole body	3

### Total RNA isolation and cDNA synthesis

Total RNA was extracted using TRIzol reagent (Ambion, Life Technologies, USA) following the manufacturer’s instructions. A NanoDrop2000 (Thermo Scientific, USA) was used to check the concentration and quality of each RNA sample. Qualified RNAs (A260/280: 1.9 to 2.1) were used for further cDNA synthesis with a First Strand cDNA Synthesis Kit (Takara, Tokyo, Japan). The cDNA of all samples was stored at -20°C before use.

### Reference gene selection and primer design

The involved reference gene sequences were downloaded from NCBI (http://www.ncbi.nlm.nih.gov/). The candidate primers were designed by the online primer design tool for real-time PCR (http://www.idtdna.com/site) with default settings. The primers were synthesized by Tsingke Biotech (Wuhan, China). The length and identity of PCR products were assessed by gel electrophoresis and sequence analysis. Then, the sensitivity, specificity, and capacity of the correct primers were tested by the melting curve and standard curve with the Bio-Rad CFX96/384 Real-Time System. Finally, the remaining qualified primers were used for further qPCR evaluation. A standard curve was achieved for each gene by five-fold serial dilution of the template.

### Quantitative real-time PCR (qPCR)

The qPCR reaction system (10 μl) contained 2.6 μl ddH_2_O, 5.0 μl TB Green MasterMix (NEWBIOTech., Canada), 0.4 μl of forward and reverse primers (10 μM) and 100 ng first-strand cDNA. The PCR program for all the genes included an initial denaturation step for 30 s at 95°C, followed by 40 cycles at 95°C for 5 s and 60°C for 30 s. Finally, a melting curve analysis from 65°C to 95°C was performed to confirm the specificity of the PCR products. Three technical replicates were analysed for each biological replicate.

### Stability of gene expression

The stability of the eight candidate reference genes was comprehensively evaluated using RefFinder (https://www.heartcure.com.au/reffinder). RefFinder is a web-based analysis tool that integrates all four major computational programs, including geNorm [30], NormFinder [31], BestKeeper [32], and the ΔCt method [33]. All of these methods can recommend the most stable reference genes; geNorm can also calculate the pairwise variation (V) of one gene by comparing values with other genes. Finally, geNorm suggests a minimum number of reference genes by V value for normalization. If Vn/n+1 is less than 0.15, no more reference genes are needed for normalization [30].

### Reference gene validation

Researches showed that the expression of heat shock protein 90 (*Hsp90*) in organisms will increase under heavy metal stress [34–36], so *Hsp90* was selected as a target gene to evaluate the stability of candidate reference genes under different heavy metals. The normalization factors (NFs) were computed based on the geometric mean of genes with the lowest Geomean values as determined by RefFinder and a single reference gene with the lowest or highest Geomean value. The relative expression level of *Hsp90* under heavy metal exposure was calculated using the 2^−ΔΔCt^ method. Data were statistically analyzed through IBM SPSS Statistics 20.0 software. Differences among the experimental results were analyzed by one-way ANOVA (Tukey’s test), p < 0.05 was considered significant.

## Results

### Expression profiles of reference genes

**Fig 1:**
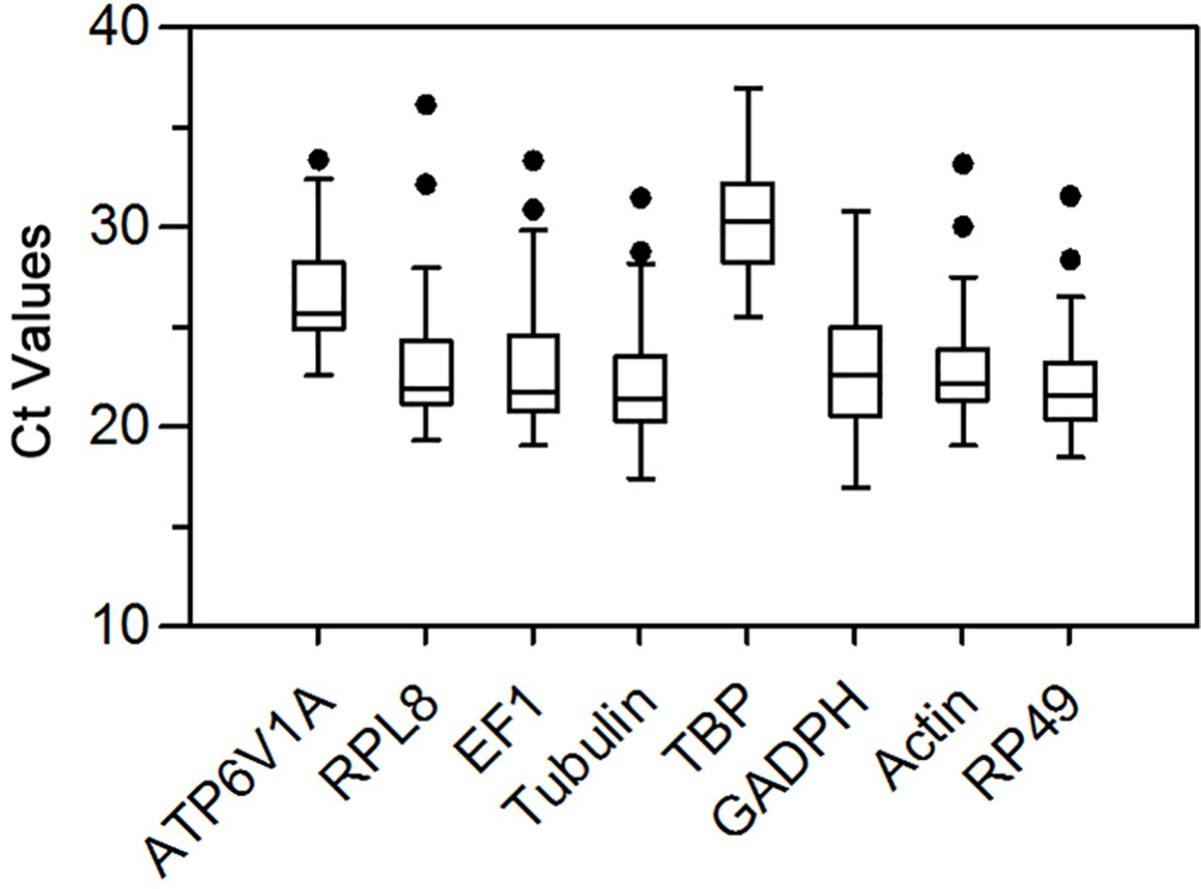
Ct values of candidate reference genes in total samples of *H*. *illucens*. The data are presented as box-whisker plots. The box represents 25th-75th percentiles. The median is represented by a bar across the box; whiskers on each box are determined by Tukey’s test; and the black spots refer to extreme outliers.

A single amplicon was designed for each candidate reference gene, and the specificity was evaluated by agarose gel electrophoresis analysis and melting curve analysis. Qualified primers used for qPCR, their PCR efficiency and regression coefficient are shown in Table 2. The efficiency values of all primers ranged between 93.2 and 108.6%, with regression coefficient values from 0.978 to 0.999, which confirmed the standard [37]. Expression profiles of the eight candidate reference genes were calculated within the selected experimental conditions (Fig 1). Raw Ct values of reference genes ranged from 16.96 (*GAPDH*) to 36.95 (*TBP*), while most Ct values were between 19 and 26. Among the genes, *RPL8* (22.86), *Actin* (22.94) and *RP49* (22.10) were the most abundant transcripts, reaching the threshold fluorescence peak after 23 cycles. The expression level of *TBP* (30.21) was the lowest.

**Table 2:** Primers of the candidate qPCR reference genes.

Gene	Accession Number	Primer Sequence (5’-3’)	Amplicon Length (bp)	PCR Efficiency	Regression Coefficient
*Actin*	MK210584	F: CGTAGGAGACGAAGCACAAA	104	0.952	0.999
		R: GGTGCCAGATCTTCTCCATATC			
*Tubulin*	MK210585	F: GCTCTCTACGACATCTGCTTTA	101	0.933	0.999
		R: CAGGTGGTAACTCCAGACATT			
*RPL8*	MK210586	F: GCCGTGCATACCACAAATAC	130	0.932	0.996
		R: TTGACTGTCGAAGCCTTACC			
*GAPDH*	MK210587	F: CCAACGTATCTGTCGTTGACT	107	0.941	0.999
		R: AATTCCCTTGAGTGGTCCTTC			
*RP49*	MK210588	F: CCCACTGGCTTCAAGAAGTT	102	0.943	0.999
		R: CGAAACTCCATGTGCGATCT			
*EF1*	MK210589	F: CGAGAAGGAAGCCCAAGAAA	116	1.086	0.978
		R: CGAACTTCCACAGGGCAATA			
*ATP6V1A*	MK210590	F: CATGGCCACTATTCAGGTCTAC	107	0.951	0.999
		R: CCATAATACCTGGACCCAACTC			
*TBP*	MK210591	F: ACCGAAAGATGCCGTGAAT	101	0.987	0.989
		R: TACGAGTGCGGAAGTTGATG			
*Hsp90*	KY457332.1	F: GTGCCAAACTCGCTGATTTC	104	0.967	0.997
		R: GTGCTTCTGGTTCTCCTTCA			

### Stability of reference genes under biotic conditions

**Fig 2:**
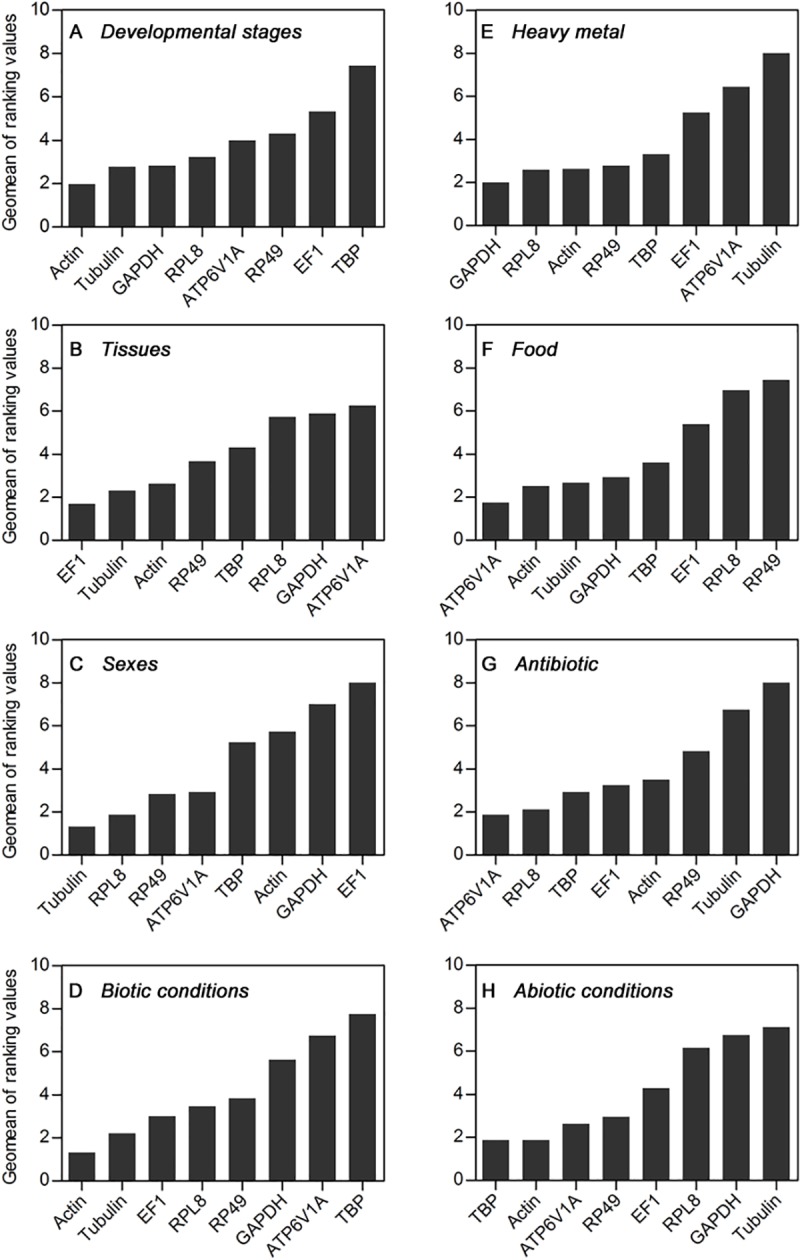
Comprehensive gene stability analysis of candidate reference genes in H. illucens. The stability of reference genes was calculated by RefFinder. A lower Geomean value indicates more stable expression. Expression stability is listed in the following samples: A-developmental stages; B-tissues; C-sex; D-biotic conditions; E-heavy metals; F-food; G-antibiotics; and H-abiotic conditions.

For different developmental stages, NormFinder ranked stability from high to low as follows: *Actin*, *RPL8*, *Tubulin*, *GAPDH*, *EF1*, *ATP6V1A*, *RP49*, and *TBP*; BestKeeper provided the ranking as *RP49*, *Tubulin*, *RPL8*, *GAPDH*, *Actin*, *TBP*, *ATP6V1A*, and *EF1*; geNorm calculated the ranking sequence as *ATP6V1A* = *GAPDH*, *Actin*, *EF1*, *Tubulin*, *RPL8*, *RP49*, and *TBP*; and the ΔCt ranking was *Actin*, *Tubulin*, *RPL8*, *GAPDH*, *EF1*, *ATP6V1A*, *RP49*, and *TBP* (Table 3). By synthesizing the results of four programs, RefFinder recommended *Actin*, *Tubulin* and *GAPDH* as the top three candidate reference genes in different developmental stages. *Actin* was the most stable reference gene, while TBP was the least stable reference gene (Fig 2A).

**Table 3:** Ranking order of candidate reference genes in response to biotic conditions.

Biotic conditions	Reference genes	NormFinder		BestKeeper		geNorm		ΔCt	
		Stability	Rank	Stability	Rank	Stability	Rank	Stability	Rank
Developmental stages	*ATP6V1A*	1.454	6	2.269	7	1.087	1	1.796	6
	*RPL8*	0.786	2	1.753	3	1.354	6	1.521	3
	*EF1*	1.382	5	2.46	8	1.17	4	1.76	5
	*Tubulin*	0.79	3	1.671	2	1.234	5	1.517	2
	*TBP*	1.964	8	2.209	6	1.73	8	2.242	8
	*GAPDH*	0.903	4	1.876	4	1.087	1	1.55	4
	*Actin*	0.483	1	1.928	5	1.106	3	1.384	1
	*RP49*	1.75	7	1.3	1	1.559	7	2.0687	7
Tissues	*ATP6V1A*	2.143	8	2.387	3	1.715	8	2.387	8
	*RPL8*	1.534	6	2.788	6	1.223	5	1.848	6
	*EF1*	0.768	2	2.338	2	0.751	1	1.467	2
	*Tubulin*	0.514	1	2.809	7	1.144	4	1.381	1
	*TBP*	1.623	7	2.299	1	1.491	7	1.995	7
	*GAPDH*	1.095	5	3.209	8	1.298	6	1.628	5
	*Actin*	0.848	3	2.467	4	0.751	2	1.509	4
	*RP49*	0.872	4	2.712	5	1.073	3	1.504	3
Sex	*ATP6V1A*	0.178	3	0.193	2	0.265	4	0.393	3
	*RPL8*	0.158	2	0.212	3	0.215	1	0.379	2
	*EF1*	0.637	8	0.656	8	0.465	8	0.672	8
	*Tubulin*	0.121	1	0.133	1	0.239	3	0.371	1
	*TBP*	0.23	5	0.373	6	0.302	5	0.416	5
	*GAPDH*	0.635	7	0.407	7	0.396	7	0.668	7
	*Actin*	0.25	6	0.349	5	0.317	6	0.419	6
	*RP49*	0.2	4	0.307	4	0.215	1	0.403	4

RefFinder showed that *EF1*, *Actin* and *Tubulin* were the three foremost reference genes in different tissues. Furthermore, *EF1* was categorized as the most stable reference gene, whereas *ATP6V1A* was the least stable reference gene (Fig 2B). RefFinder ranked three different reference genes, including *Tubulin*, *RPL8* and *RP49*, in different sexes. Among these genes, *Tubulin* was the most stable reference gene. In contrast, the reference gene *EF1* was found to be the least stable reference gene (Fig 2C). Based on the above three biotic conditions, RefFinder provided a general ranking of *Actin*, *Tubulin*, *EF1*, *RPL8*, *RP49*, *GAPDH*, *ATP6V1A*, and *TBP* (Fig 2D). According to the results of the four programs (NormFinder, BestKeeper, geNorm and ΔCt), the stability of eight candidate reference genes in response to biotic conditions is shown in Table 3.

### Stability of reference genes under abiotic conditions

For different heavy metals, *GAPDH*, *RPL8* and *Actin* were the top three reference genes according to the comprehensive ranking from RefFinder. Specifically, *GAPDH* and *Tubulin* were the most and the least stable reference genes, respectively (Fig 2E). For different food sources, *ATP6V1A*, *Actin* and *Tubulin* were the top three reference genes according to the comprehensive ranking from RefFinder. *ATP6V1A* and *RP49* were the most and the least stable reference genes, respectively (Fig 2F). For different antibiotics, *ATP6V1A*, *RPL8* and *TBP* were the top three reference genes according to the comprehensive ranking from RefFinder. *ATP6V1A* and *GAPDH* were the most and the least stable reference genes, respectively (Fig 2G). Based on these three abiotic conditions, RefFinder offered a general ranking of *TBP*, *Actin*, *ATP6V1A*, *RP49*, *EF1*, *RPL8*, *GAPDH*, and *Tubulin* (Fig 2H). According to the results of four programs (NormFinder, BestKeeper, geNorm and ΔCt), the stability of eight candidate reference genes in response to abiotic conditions is shown in Table 4.

**Table 4:** Ranking order of candidate reference genes in response to abiotic conditions.

Abiotic conditions	Reference genes	NormFinder		BestKeeper		geNorm		ΔCt	
		Stability	Rank	Stability	Rank	Stability	Rank	Stability	Rank
Antibiotics	*ATP6V1A*	0.418	1	0.847	4	0.903	3	1.191	1
	*RPL8*	0.679	2	0.967	5	0.826	1	1.239	2
	*EF1*	0.857	4	1.175	7	0.826	1	1.339	4
	*Tubulin*	1.523	7	1.097	6	1.297	7	1.781	7
	*TBP*	0.682	3	0.717	2	1.012	4	1.275	3
	*GAPDH*	1.695	8	1.24	8	1.454	8	1.925	8
	*Actin*	1.012	5	0.522	1	1.132	6	1.434	5
	*RP49*	1.065	6	0.745	3	1.092	5	1.449	6
Food	*ATP6V1A*	0.74	3	1.603	1	0.455	1	1.76	3
	*RPL8*	1.913	7	2.946	8	1.121	6	2.347	7
	*EF1*	0.983	6	2.393	7	0.897	5	1.782	4
	*Tubulin*	0.788	5	1.667	2	1.302	7	1.791	5
	*TBP*	0.311	1	1.933	4	0.455	1	2.082	6
	*GAPDH*	0.77	4	1.834	3	0.657	3	1.706	2
	*Actin*	0.579	2	2.026	5	0.845	4	1.697	1
	*RP49*	5.292	8	2.329	6	2.314	8	5.35	8
Heavy metals	*ATP6V1A*	0.57	7	0.731	5	0.598	7	0.703	7
	*RPL8*	0.471	5	0.624	3	0.249	1	0.62	3
	*EF1*	0.412	3	0.836	7	0.533	6	0.623	6
	*Tubulin*	0.647	8	0.941	8	0.635	8	0.746	8
	*TBP*	0.407	2	0.808	6	0.464	5	0.614	2
	*GAPDH*	0.218	1	0.688	4	0.429	4	0.535	1
	*Actin*	0.436	4	0.51	1	0.379	3	0.62	4
	*RP49*	0.48	6	0.529	2	0.249	1	0.621	5

### Optimal number of reference genes

**Fig 3:**
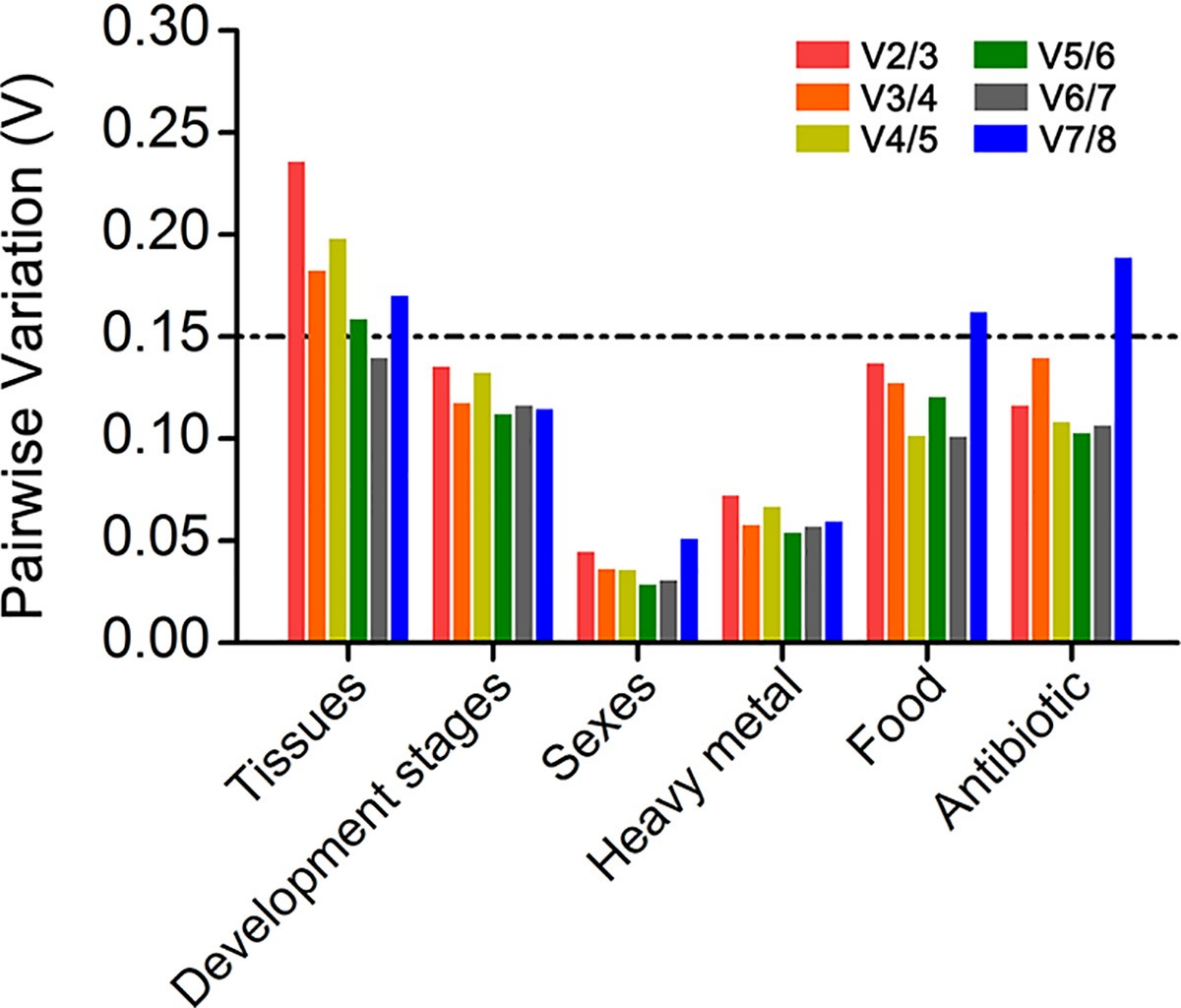
Optimal number of reference genes for normalization under different experimental conditions. The pairwise variation (V_n/n+1_) was analysed by geNorm software to determine the optimal number of reference genes. Values less than 0.15 suggest that another reference gene will not be required for the normalization of gene expression.

The optimal number of reference genes was determined using geNorm by the paired variation (Vn/n+1) value. If Vn/n+1 was less than 0.15, it was unnecessary to apply an additional reference gene, i.e., top-ranking n reference genes were sufficient to use as control genes. Based on the comprehensive ranking (Fig 2) and paired variation value (Fig 3), reference genes under each experimental condition were determined. The results of the geNorm analysis showed that the value of V2/3 was < 0.15 under different developmental stages. Therefore, *Actin* and *Tubulin* were the most appropriate reference genes. For different tissues, the recommended reference genes were *EF1*, *Tubulin*, *Actin*, *RP49*, *TBP* and *RPL8*. In different sexes, *Tubulin* and *RPL8* were recommended. *GAPDH* and *RPL8* were considered the most appropriate reference genes for different heavy metals. The analysis of geNorm predicted that the values of V2/3 were < 0.15 both in different food and different antibiotic treatments, which suggested that *ATP6V1A* and *Actin* were the best combination of reference genes under different food conditions, while *ATP6V1A* and *RPL8* were the most suitable for *H*. *illucens* under different antibiotic conditions.

### Reference genes validation

**Fig 4:**
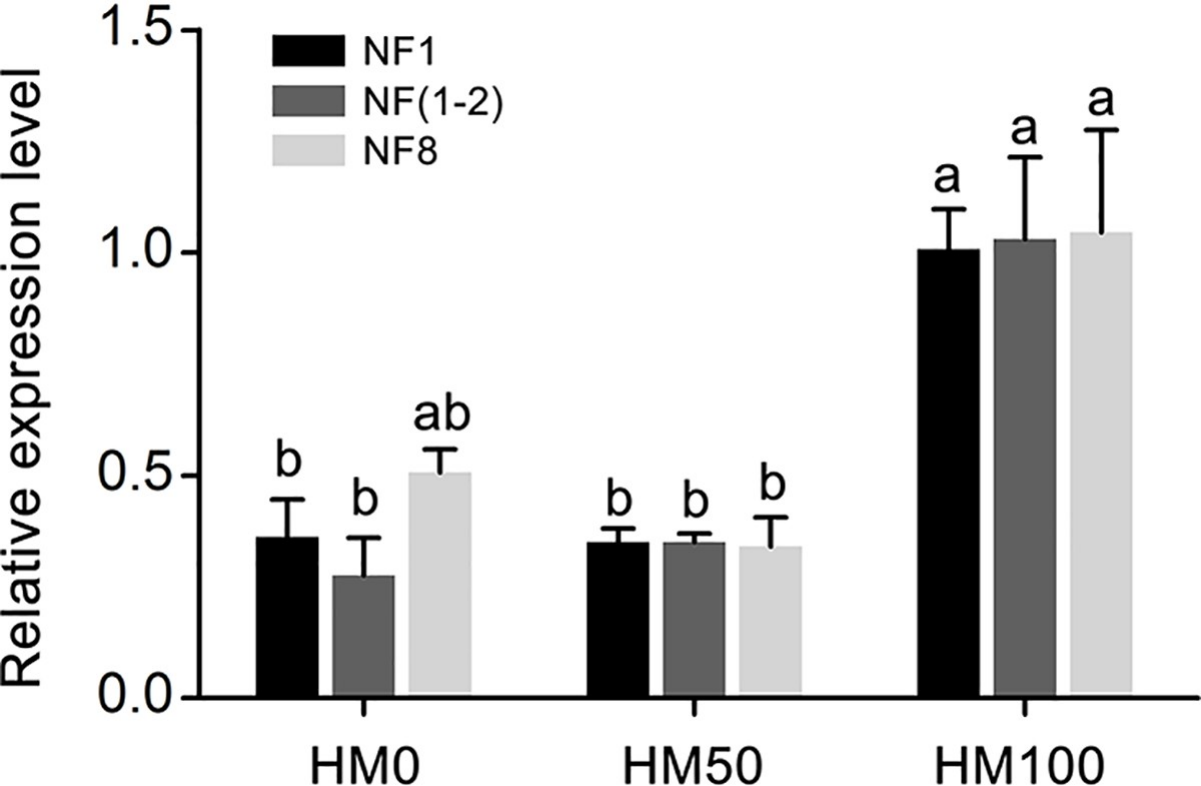
Validation of reference gene stability. The expression levels of *Hsp90* under different heavy metal conditions were identified using different reference genes. Data represent the mean values ± SEM of three biological replicates. The different letters in the same color columns indicate significant differences among treatments within each normalization factor (*p* = 0.05, Tukey’s HSD test).

*Hsp90* was selected as a target gene to validate the accuracy of the identified reference gene under different heavy metal conditions. The expression of *Hsp90* was calculated by using the most stable reference gene *GAPDH* (NF1), the top two stable reference genes *GAPDH* and *RPL8* (NF1-2), and the least stable gene *Tubulin* (NF8) for normalization (Fig 4). The expression profile showed a similar trend in both NF1 and NF1-2. When using NF1 and NF1-2 as reference genes, the expression of *Hsp90* was not significantly different in 0 mg/kg of cadmium (HM0) and 50 mg/kg of cadmium (HM50), and the expression in HM0 was significantly lower than that in 100 mg/kg of cadmium (HM100). In comparison, when NF8 was used as a reference gene, *Hsp90* expression was no significant difference between HM0 and HM100 (Fig 4).

## Discussion

Because of its high sensitivity, specificity, rapidity and reliability, qPCR is considered the most suitable method for the quantification of gene expression [38]. Reliable reference genes are particularly important in eliminating heterogeneity in various datasets and ensuring the accuracy of quantitative results [39]. A gene that maintains a constant expression level under different experimental conditions is considered an ideal reference gene. However, not all reference genes have this characteristic in different species. According to Bustin et al. [38], the stability of reference genes has been studied in many insect species [23, 26, 27]. As a potential biomaterial for the conversion of waste, the stability of reference genes of *H*. *illucens* plays an important role in understanding molecular mechanisms. To the best of our knowledge, this is the first report on the identification of appropriate reference genes for qPCR analysis in *H*. *illucens*.

In this study, we evaluated the stability of eight candidate reference genes for *H*. *illucens* gene expression analysis under six experimental conditions (developmental stages, tissues, sex, heavy metal, food, and antibiotics). The first three biotic conditions have been used to evaluate the stability of reference genes in many insects [26, 27, 40]. The latter three abiotic conditions were selected according to the high frequency of their occurrence as experimental treatments in the study of *H*. *illucens*. First, as a saprophytic insect, *H*. *illucens* can transform a wide range of organic wastes, most of which are faeces and waste food [41–43]. As a control diet [44], wheat bran was also analysed in this study. Second, due to exposure to heavy metals and antibiotics in the environment (especially in pig manure), there have been many studies on heavy metals and antibiotics in *H*. *illucens* [45–47].

The stability ranking of reference genes was different between the different experimental conditions. For example, *Actin* was the most stable reference gene in the developmental stages, while it was the third most unstable gene in the different sexes. *TBP* was the most stable reference gene in all abiotic conditions and the least stable in biotic conditions (Fig 2). Despite some differences in individual rankings, *Actin* was consistently maintained at a higher level of stability than the rest of the reference genes (Fig 2). Previous results showed *Actin* to be an optimized reference gene in *Drosophila melanogaster*, *Bemisia tabaci*, *Chilo suppressalis* and *Bombyx Mori* [48–51]. In summary, some genes can be used as reference genes under many experimental conditions, but the best way to find suitable reference genes is to evaluate their stability under specific experimental conditions.

Recently, a single gene has been replaced by multiple genes to normalize the expression level of target genes in qPCR analyses [52]. Traditionally, the optimal number of reference genes is customarily determined by geNorm [30]. In this study, our results not only ranked the stability of genes under different experimental conditions but also provided the optimal number of reference genes to improve the accuracy of qPCR analysis (Fig 2 and Fig 3). Two reference genes were recommended for different developmental stages (*Actin* and *Tubulin*), sex (*Tubulin* and *RPL8*), heavy metals (*GAPDH* and *RPL8*), food (*ATP6V1A* and *Actin*) and antibiotics (*ATP6V1A* and *RPL8*), while six reference genes were required for reliable normalization in tissues (*EF1*, *Tubulin*, *Actin*, *RP49*, *TBP* and *RPL8*). However, our recommended reference genes were selected from eight candidate reference genes, which does not mean that other candidate genes cannot be used. It would be more appropriate for potential audiences to verify these results flexibly than to apply them directly.

In conclusion, this study screened a series of reference genes for future work on gene expression in *H*. *illucens* and provides a solid foundation for further molecular biology research to understand how this important resource insect copes with harsh environmental conditions.

## Supporting information

S1 Table DATA used to build Fig 1. (XLSX)

S2 Table DATA used to build Fig 2. (XLSX)

S3 Table DATA used to build Fig 3. (XLSX)

S4 Table DATA used to build Fig 4. (XLSX)
